# Rubefacients and Vesicants

**Published:** 1895-07

**Authors:** A. W. Harlan

**Affiliations:** Chicago, Ill.


ARTICLE VII.

### RUBEFACIENTS AND VESICANTS.

BY A. W. HARLAN, M. D., D. D. S., CHICAGO, ILL.

**Read at the Union Meeting of the Fifth, Sixth, Seventh, and Eighth District Dental Societies, held in Buffalo, October, 1894.**

When I was urged to present a short paper to the members of this gathering, I did not fully make up my mind to write upon the above topics, as I had something more practical on hand, but after a time I concluded that occasional practicability was all that was needed to make some subjects interesting.

You are asking of what value to dentistry a consideration of reddening and blistering will be to busy practitioners? I will try and answer your question. There are many conditions requiring the use of a rubefacient, and not a few where a blister is of immense value. It is not necessary, and it would defeat the effect of these brief notes, if an attempt was made to write a full paper on counter-irritation. My opinion is that rubefaction and vesication, as remedial measures, are not taken advantage of by dentists sufficiently often to make them familiar with their objects in medicine.

What is a rubefacient? This is the definition of Dunglison: "Rubefacient; producing redness, a medicine which produces redness of the skin. The action is called rubefaction, a gentle, local irritant. Vesicant; a blister, vesication, the action of a vesicant, the formation of blisters, vesicating blister, epispastic. Blister; vesicle or vesication, from vesicatories or other causes, also vesicant, vesicating; a blister plaster, substance which when applied to the skin irritates it and occasions serous secretion, raising the epidermis and inducing vesicles, as cantharides, mustard ammonia, etc. Blisters are used as counter irritants. A blister applied for a few hours to produce this effect is termed a fly-blister. A perpetual blister is one kept open by appropriate dressing." The latter definition is also from Dunglison. Perhaps the simplest case of dental disease is pulpitis. Of what value would rubefaction be to the patient? Local anodynes and even local anæsthetics often fail to give relief, and the pulp is doomed to die from lack of appropriate measures. In many cases of this nature the application of hot water to the neck and above the ear on the affected side, by means of a few thicknesses of heavy towelling (six or eight thicknesses) soaked and partially wrung out, will prove efficacious, if continued from five to ten minutes. In nearly all cases of pain around erupting third molars, the use of water about 120° F. to 130° F., covering an area of from four to six inches from the focal point, will give the necessary relief in a few minutes. The direct application of a small jet of water in the mouth on the inflamed surface is both painful and injurious, as gentle unloading of the engorged vessels is not accomplished in this manner.

For minor ills of the gums (in pyorrhœa), rubefaction may be accomplished without vesication by using a saturated solution of menthol in alcohol, or even less than this, down to a ten-per-cent. solution. Oil of peppermint, oil of turpentine or oil of cloves will produce reddening, and when used over a large area will often so alter the blood-current that there will not be anything more than swelling without suppuration. Rubefaction may be produced with chloroform, camphor, and aqua ammonia. Mustard and capsicum are also used for this purpose. It must be remembered that the object of rubefaction is to draw the blood from a circumscribed inflamed area, and fill other unstimulated points, giving nature an opportunity to recover. I have been surprised and grieved to find that rubefaction is practised on too small a territory, not sufficient to give the desired relief. Estimated that an inflamed spot is one-half an inch square, the rubefacient should cover ten times this area *in a locality which will direct the flow of the blood elsewhere* and relieve the tension on the arteries or arterioles. In acute pericementitis, in addition to rubefaction, it may be necessary to produce vesication—blistering. Many agents are used for this purpose, none of them very nicely or in appropriate places. A blister to relieve pericementitis in a superior central incisor should be made over the roots of the bicuspids, and the gingival margin of the gum, around the central incisor, should be painted at the same time with compound tincture of iodine. The blood-supply will be deflected and the resolvent effect of the iodine will soon be felt around the apex of the root The gum must receive at least two paintings. This rule applied to all such cases will give much cause for congratulation from your clients. Ammonia, capsicum, cantharides or black mustard oil will produce blisters. Carbolic acid will produce a poor blister. Do not use zinc chloride for this purpose. A blister will do no good after the formation of pus around the apex of the root. Rubefaction may do something to alleviate the pain, but a blister increases it. Rubefaction and blistering for inflammation of the gums and the peridental membrane, facial neuralgia, as well as the pulp of a tooth, will be more efficient than leeching and purging

A modification of Mayer's hammer may be used with good effect for rubefaction. Mayer's hammer is a steel disk dropped in boiling water before it is used on the mucous membrane or the skin. The one I use is about the size of a copper cent, and twice as thick, with a handle that is screwed into it while it is in the water; by applying this gently over three or four inches of space, great reddening is produced.

What the patient needs is a new sensation; this he gets with a blister or a rubefacient. When you are about to produce rubefaction or vesication, do it with your whole soul. Do it well; do not be afraid. Use blisters in inflammation, and rubefacients in congestion or stagnation. An ugly blister is not needed, as it will cause sloughing, and this is unnecessary around the mouth or on the face.—*Dental Practitioner and Advertiser.*

